# Unguentum Glycerini, &c., &c.

**Published:** 1860-04

**Authors:** 


— Dr. Pierce observes that he has seen stramonium ointment, as well as the whole list of preparations used for excoriated nipples, but no one has been so universally followed by good results as that made after the following: Tannic acid, gr. xx.; glycerine; alcohol, ää, ℥j. M.

— In 1838, England, with a population of 15,000,000, consumed 35,000,000 pounds of tea; in 1858, with a population of nearly 18,000,000, about 81,000,000.

— Our associate, *Prof. Austin Flint, Jr.*, has recently been appointed to the Chair of Physiology and Microscopic Anatomy in the New Orleans School of Medicine. The former incumbent of this Chair, Prof. Peniston, has been transferred to the Chair of Anatomy, Prof. Beard resigning it and assuming the duties of the Chair of the Principles of Surgery and Surgical Pathology, while Prof. Choppin takes a new Professorship, to be called the Chair of Clinical and Operative Surgery. Dr. Flint, previous to accepting this position, had resigned his Professorship in the New York Medical College.

